# Supplementary material for: How to make sense of information about COVID-19 and the vaccine from the authorities? A qualitative study of migrants’ experiences in two Swedish communities
Source: PLOS Glob Public Health. 2025 Dec 10;5(12):e0004489. doi: 10.1371/journal.pgph.0004489 (PMC12694806; doi:10.1371/journal.pgph.0004489)
Supplement: S1 Text — (DOCX) [file pgph.0004489.s001.docx]

**Interview questions**

How did you get information about COVID-19?

Where did you get information about COVID-19?

Did information channels change over time?

Have there been difficulties in orientating yourself about COVID-19?

Have you experienced conflicting information?

What information has been most useful?

What information have you missed?

How have you experienced information given in Swedish?

How have you experienced the information given in your mother tongue?

Have you been in contact with the telephone line in different languages?

- If so, how did you feel about the information you received?

Have you been in contact with health information providers in the local area?

How would you like to be informed in case of a future pandemic?

How did you receive information about the COVID-19 vaccine?

Where did you receive information about vaccination against COVID-19?

Have you experienced difficulties in orientating yourself about vaccination against COVID-19?

Have you experienced conflicting information about vaccination?

What information about vaccination has been most useful?

What information about vaccination have you missed?

How have you experienced information about vaccination given in Swedish?

How have you experienced the information about vaccination given to you in your mother tongue?

How would you like information about vaccination to be provided in the event of a future pandemic?

What can create trust (besides language, perhaps religion, life experience, age, gender, etc.) in the person giving information in similar situations?

Other comments?
