# Supplementary material for: How to make sense of information about COVID-19 and the vaccine from the authorities? A qualitative study of migrants’ experiences in two Swedish communities
Source: PLOS Glob Public Health. 2025 Dec 10;5(12):e0004489. doi: 10.1371/journal.pgph.0004489 (PMC12694806; doi:10.1371/journal.pgph.0004489)
Supplement: Attachment — Submitted filename: Response to Reviewers.docx [file pgph.0004489.s003.docx]

To PLOS Global Public Health

Dear Editor Dr Myung-Bae Park

We have now revised the manuscript “PGPH-D-25-00670 How to make sense of information about COVID-19 and the vaccine from the authorities? A qualitative study of migrants' experiences in two Swedish communities” according to the comments of the editor and reviewers.

Our revision includes:

1. A rebuttal letter that responds to each point raised by the editor uploaded as a separate file labelled 'Response to Reviewers'.

2. A marked-up copy of our manuscript that highlights changes made to the original version as a separate file labelled 'Revised Manuscript with Track Changes'.

3. An unmarked version of our revised paper without tracked changes labelled 'Manuscript'.

Best regards,

Sofie Bäärnhielm

**Journal Requirements:**

1. Please provide additional details regarding participant consent. In the ethics statement in the Methods and online submission information, please ensure that you have specified (1) whether consent was informed and (2) what type you obtained (for instance, written or verbal, and if verbal, how it was documented and witnessed).

Our response

We have now provided additional information about participant consent by moving the sentence about written consent from the heading “Ethics and preregistration” to “Sampling and data collection” and added this text:

*“Written and oral information about the study was given to the participants, they were informed that they could withdraw participation without motivation. Written consent was obtained by the interviewer and was stored separately from the interviews according to the data regulations of Region Stockholm. Sampling did not take place within the framework of healthcare contacts.”*

2. Please provide additional information regarding the considerations made for the migrants included in this study. For instance, please discuss whether participants were able to opt out of the study and whether individuals who did not participate receive the same treatment offered to participants.

Our response

Concerning whether participants were able to opt out, please see our response to Reviewer 1 comment 1 and Reviewer 2 about sample composition and characteristics.

Although this study was conducted with the healthcare sector as the authority responsible for the study, it was never based on a healthcare contact and accordingly had no implications for treatment. We have clarified this with this sentence:

*“Sampling never took place within the framework of healthcare contacts.”*

3. We have amended your Competing Interest statement to comply with journal style. We kindly ask that you double check the statement and let us know if anything is incorrect.

Our response:

We have no competing interests

4. In the online submission form, you indicated that “Availability of data and materials

The datasets used during the current study are available from the corresponding author on reasonable request.”.

All PLOS journals now require all data underlying the findings described in their manuscript to be freely available to other researchers, either

a. In a public repository,

b. Within the manuscript itself, or

c. Uploaded as supplementary information.

This policy applies to all data except where public deposition would breach compliance with the protocol approved by your research ethics board. If your data cannot be made publicly available for ethical or legal reasons (e.g., public availability would compromise patient privacy), please explain your reasons by return email and your exemption request will be escalated to the editor for approval. Your exemption request will be handled independently and will not hold up the peer review process, but will need to be resolved should your manuscript be accepted for publication. One of the Editorial team will then be in touch if there are any issues.

Our response

Our data cannot be made publicly available for ethical and legal reasons as this would compromise participants’ privacy and breach compliance with the protocol approved by our research ethics board. The study was approved by the Swedish Ethical Review Authority (No. 2022-01637-01). We have sent a separate return email about exemption request.

Due to data availability, we have contacted the journal editors, as ethical and legal restrictions prevent us from sharing the deidentified dataset. [EMID:382252684e0aaf09]

We have included this text in the Data Availability statement:

The data that we can share includes the study protocol, which is preregistered on the Open Science Framework (osf.io/rt47j), as well as the interview guide and code tree, which can be found at https://doi.org/10.5281/zenodo.17389414.

For data request you can contact associate professor Anna Clara Hollander, PhD, Karolinska Institutet, Department of Global Health. email: anna-clara.hollander@ki. Dr. Hollander has no relationship to the present study.

5. If the reviewer comments include a recommendation to cite specific previously published works, please review and evaluate these publications to determine whether they are relevant and should be cited. There is no requirement to cite these works unless the editor has indicated otherwise.

Our response

We have got now recommendations to cite specific previously published works.

6. Please review your reference list to ensure that it is complete and correct. If you have cited papers that have been retracted, please include the rationale for doing so in the manuscript text, or remove these references and replace them with relevant current references. Any changes to the reference list should be mentioned in the rebuttal letter that accompanies your revised manuscript. If you need to cite a retracted article, indicate the article’s retracted status in the References list and also include a citation and full reference for the retraction notice.

Our response:

We have changed one reference number 16 by including this recently published reference:

*Eklund SM, Bäärnhielm S, Al-Ammari B, Abshir H, Strand M, Cetrez ÖA and Puthoopparambil SJ (2025) Experiences of health informers working with intercultural communication in Stockholm, Sweden during COVID-19: a qualitative study. Front. Commun. 10:1645922. doi: 10.3389/fcomm.2025.1645922*

**Response to reviewer 1**

Reviewer #1: This paper examines how immigrant communities understood the Swedish government's COVID communications. The authors highlight several challenges, particularly language barriers, mistrust of the government, religion etc as some of the issues that migrants struggled with. The authors interviewed 76 individuals, of varying ages. Overall, the paper is clearly written although it is extremely long and could benefit from being shorter so that the key points being made are easier to grasp. The discussion, in particular, is much too long even for a qualitative paper. In terms of methodological rigor, the study is well designed and it is clear that the authors collected a lot of information. It also helps that some of the authors could speak some of the languages represented by the migrant communities they interviews.

Our response

Thanks for this comment. We have now extensively shortened the manuscript, in particular the discussion section but also the introduction and some parts of the results section.

**Response to reviewer 2**

Reviewer #2: A study addressing the experiences of migrants and linguistic minority groups during the global health crisis of the COVID-19 pandemic provides an important message to the international academic community. By interviewing 76 migrants with diverse ages, genders, and language backgrounds, the study successfully captures multiple perspectives.

However, the manuscript would become a more meaningful contribution if the following points are addressed:

Sample composition: Detailed information on participants’ socioeconomic backgrounds (e.g., education, occupation, length of residence) is limited. Clarifying how these background variables influence the understanding of COVID-19 information would strengthen the credibility of the analysis. In the same vein, indicating the interviewees’ age, gender, and language background alongside quotations would help readers better understand the context.

Our response:

Though additional personal characteristics would be very useful for further analysis, we have not collected these for ethical reasons. This would highly increase the risk of recognition and back-tracking, as these communities are small and vulnerable. There was already an infected and stigmatized political, social and media discussion, which necessitated us to be careful in field work and when approaching our participants. There is currently also a stigmatizing discussion in Sweden about migrants’ right to remain in the country, and about the authorities trying to expel those who do not have permission to stay and questioning permissions to stay. This is an especially important issue in the two areas where our study are being conducted. As a researcher, it is very easy to find oneself in a situation where the community becomes closed off.

However, we can add gender and age group. We have chosen not to add the age group to the quotes from focus groups, as this facilitates tracking individuals back.

We have added this new text under the heading Ethics and preregistration

*“The communities involved are in a vulnerable situation. Given their small size, careful considerations in fieldwork and data presentation are needed. Furthermore, at the time of the pandemic, there was an infected and stigmatized political, social, and media discourse, which required a careful and responsible approach to fieldwork from our side. For these reasons we have not provided any personal characteristics when presenting the results, as this would highly increase the risk of recognition and back-tracking.”*

**Reviewers comment**

Policy implications: While the conclusion highlights the importance of community preparedness, it would be valuable to specify concrete implementation strategies (e.g., multilingual crisis communication manuals, training programs for health leaders, rapid translation systems) to enhance the study’s policy relevance.

Our response

Thanks for this important comment. We have changed the subheading “Implication and for future health crisis communication” to include the word recommendations and the subheading is now: “Implication and recommendations for future health crisis communication” and have added this text:

*“Suggestions on key aspects to be considered while communicating with linguistically diverse populations in future health crises*

- *Continuous analysis of existing public health communication strategies, including language needs in populations.*
- *Continuously preparing for potential health-related crises, as diversity in the population and communication channels to reach subgroups keep evolving.*
- *Anchor communication in local and community specific trust-building strategies to reach vulnerable populations.*
- *Establish partnerships with local communities, individual or institutional, as part of national public health strategies and use these partnerships as soon as possible during health crises for effective dialogue and information dissemination*
- *Be aware of power differences and admit the agency of local communities, by inviting them to prepare and involve in crisis management and communication.“*

6. PLOS authors have the option to publish the peer review history of their article (what does this mean?). If published, this will include your full peer review and any attached files.

Our response

For us it is fine with only the final paper.
